# CSA: a web service for the complete process of ChIP-Seq analysis

**DOI:** 10.1186/s12859-019-3090-0

**Published:** 2019-12-24

**Authors:** Min Li, Li Tang, Fang-Xiang Wu, Yi Pan, Jianxin Wang

**Affiliations:** 1 School of Computer Science and Engineering, Central South University, Changsha, China; 2 Division of Biomedical Engineering and Department of Mechanical Engineering, University of Saskatchewan, SKS7N5A9, Saskatoon, Canada; 3 Department of Computer Science, Georgia State University, GA30303, Atlanta, USA

**Keywords:** ChIP-seq, Quality control, Peak calling, Downstream analysis, Visualization

## Abstract

**Background:**

Chromatin immunoprecipitation sequencing (ChIP-seq) is a technology that combines chromatin immunoprecipitation (ChIP) with next generation of sequencing technology (NGS) to analyze protein interactions with DNA. At present, most ChIP-seq analysis tools adopt the command line, which lacks user-friendly interfaces. Although some web services with graphical interfaces have been developed for ChIP-seq analysis, these sites cannot provide a comprehensive analysis of ChIP-seq from raw data to downstream analysis.

**Results:**

In this study, we develop a web service for the whole process of ChIP-Seq Analysis (CSA), which covers mapping, quality control, peak calling, and downstream analysis. In addition, CSA provides a customization function for users to define their own workflows. And the visualization of mapping, peak calling, motif finding, and pathway analysis results are also provided in CSA. For the different types of ChIP-seq datasets, CSA can provide the corresponding tool to perform the analysis. Moreover, CSA can detect differences in ChIP signals between ChIP samples and controls to identify absolute binding sites.

**Conclusions:**

The two case studies demonstrate the effectiveness of CSA, which can complete the whole procedure of ChIP-seq analysis. CSA provides a web interface for users, and implements the visualization of every analysis step. The website of CSA is available at http://CompuBio.csu.edu.cn

## Background

Next-generation sequencing technologies have produced a large amount of raw data, lots of computational methods have been developed to solve the problem of genome assembly [1–6], variation detection and annotation [7, 8], which had given rise to the release of unknown reference genome and helped interpret the complex genome structure. Based on the complete reference genome, the analysis of NGS data has become reasonable, the chromatin immunoprecipitation sequencing (ChIP-seq) [9] is an important technology for functional genomics research [10], and brought a qualitative leap for related biological experiments. The real value of the ChIP-seq technology lies not only in obtaining information about the distribution of DNA-related proteins in the genome, but also in digging deeper esoteric secrets behind such information [11].

The process of ChIP-seq contains mapping, peakcalling, and downstream analysis. Mapping is the most memory-consuming step, and lots of mapping methods are proposed to align the sequenced reads to reference genome. BWA [12] is a software package that maps low divergence sequences to a large reference genome. Bowtie [13] is a short read aligner, which is ultrafast speed and memory-efficiency. Bowtie2 [14] is used to align sequencing reads to long reference sequences, with the features of ultrafast and memory-efficiency. SOAP [15] is a faster and efficient alignment tool for short sequence reads against reference sequences. BLAST [16] is used to find the similar regions between biological sequences, which can be used to infer functional and evolutionary relationships between sequences as well as help identify members of gene families. Subread [17] also finds regions of local similarity between sequences, which aligns nucleotide or protein sequences against sequence databases and calculates the statistical significance of matches. NGM [18] has the ability to process higher mismatch rates than comparable algorithms while still performing better than them in terms of runtime, and is a flexible and highly sensitive short read mapping tool, which requires SSE enabled 64 bit dual-core. The step of peakcalling is to detect the protein modification and identify the transcription factor binding sites. MACS [19] can evaluate the significance of enriched ChIP regions by capturing the influence of genome complexity, and MACS [19] combines the information of sequencing tag positions and orientations to improve the spatial resolutions. MACS2 is an updated version of MACS [19]. PeakSeq [20] is used to identify and rank the peak regions in ChIP-Seq experiments. PeakRanger [21] takes a while for user’s browser to parse the generated HTML file. The lc tool needs about 1.7G ram per 10 million aligned reads. SICER [22] is to identify the enriched domains from histone modification ChIP-Seq data by a clustering method. The focus of Fin.

dPeaks [23] is on post-alignment analysis. This program includes interpreters for most common aligners and SNP callers and is able to use input from a wide variety of formats. Fseq [24] is to intuitively summarize and display individual sequence data as an accurate and interpretable signal. In the method of AREM [25], reads are modeled using a mixture model corresponding to K enriched regions and a null genomic background. BroadPeak [26] is abroad peak calling algorithm for diffuse ChIP-seq datasets. BCP can search the input file, and find the enrichment of peaks. PePr [27] uses a negative binomial distribution to model the read counts among the samples in the same group, and looks for consistent differences between ChIP and control group or two ChIP groups run under different conditions. The method diffReps [28] takes into account the biological variations within a group of samples and uses that information to enhance the statistical power. SISSRs [29] identifies the binding sites from short reads which are generated from ChIP-Seq experiments precisely.

In recent years, several platforms have been developed to analyze ChIP-seq experiment data. These platforms can be divided into three categories: command line, GUI, and web service. One of the most popular command line-based platform is HOMER [30], which provides NGS analysis and motif finding. ChIPseeker [31] is an R package, having both the command line and GUI version for ChIP peak annotation, comparison and visualization, while it is demands the system environment and requires installation in users’ servers. Other platforms are based on web services, such as Nebula [32] and ChIPseek [33]. Nebula integrates several peak calling methods and provides motif findings. ChIPseek is a web server based on HOMER, which also provides peak calling, motif finding and KEGG analysis. However, most of these web-based tools can neither cover the whole process of ChIP-seq analysis, nor provide the visualization of results. The downstream analysis usually includes motif finding, Gene Ontology Analysis, and pathway analysis. The algorithm findMotifs in HOMER can find the de novo motifs and known motifs. The algorithm annotatePeaks in HOMER can perform Gene Ontology Analysis, associate peaks with gene expression data, calculate ChIP-Seq tag densities from different experiments, and find motif occurrences in peaks. iPAGE [34] provides a complete meta-analysis of whole-genome datasets in cooperation with FIRE, and a *P*-value heatmap with significant categories is generated.

Here, we develop a web-based ChIP-Seq Analysis tool (CSA), which provides a comprehensive analysis of ChIP-seq data by integrating seven mapping algorithms, thirteen peak calling methods, and three downstream analysis methods. CSA places great emphasis on the workflow, which helps finish the whole analysis through several easy steps. In addition, CSA provides the visualization of the entire process. Table 1 shows a comprehensive comparison between CSA and several other typical platforms for ChIP-seq analysis including HOMER [30], ChIPSeqWorkflow [35], ChIPseeker [31], CisGenome [36], ChIP-seq tool [37], Nebula [32], and ChIPseek [33]. Table 1 also lists the systems on which the platforms rely, the installation requirement, the interface, and the functions.

**Table 1:** Current typical platforms for ChIP-Seq analysis

Platform	system	installation	Interface	Functions & tools									
				workflow	Mapping	Quality control	Peak calling	Format convert	Data visualization	Find motifs	GO analysis	Peaks annotation	Pathway analysis
HOMER[22]	UNIX LINUX MacOS	Perl installation scripts	Command line	**–**	**–**	makeTagDirectory	findPeaks	pos2bed.pl,bed2pos.pl	–	findMotifsGenome.pl	annotatePeaks.pl	annotatePeaks.pl	**–**
ChIPSeqWorkflow[27]	UNIX LINUX	compilation from source installer	Command line	–	bowtie	–	MACS	Samtools,Bedtools	–	MEME	–	–	GAGE
ChIPseeker[23]	UNIX LINUX	R package manager	GUI/Command line	√	**–**	ChIPseeqerReadCountDistribution	ChIPseeqer	–	peaks coverage	ChIPseeqerFIRE,ChIPseeqerMotifMatch	–	ChIPseeqerAnnotate	iPAGE
CisGenome[28]	no limitation	compilation from source installer (for Windows)	GUI/command line	**–**	**–**	**–**	tilemapv2	file_bed2cod,fasta_soft2hardmask, etc.	**–**	motifmap_matrixscan_genome,etc.	refgene_getnearestgene	**–**	**–**
ChIP-Seq tool[29]	no limitation/UNIX LINUX	not needed	Web server/Command line	**–**	Chipcor,Chipextract,chipscore	**–**	Chippeak, chippart	Compactsga,featreplace, etc.	**–**	**–**	**–**	**–**	**–**
Nebula[24]	no limitation	not needed	Web server	√	Bowtie	FASTQC	HMCan,MICSA,MACS,PeakSplitter,CCAT,FindPeaks	Samtools,Bamtools	**–**	ChIPmunk,AhoPro	Get peak distribution around TSS/histone	√	**–**
ChIPseek[25]	no limitation	not needed	Web server	–	–	–	HOMER	BEDTools	peak location distribution	HOMER	√	**–**	KEGG
CSA	no limitation	not needed/compilation from script	Web server/local web server	√	BWA, Bowtie, Bowtie2, SOAP,BLAST,Subread,NGM	deepTools	MACS,MACS2,PeakSeq,PeakRanger,SICER, PePr,BCP,diffReps,SISSRs, FindPeaks, AREM, Fseq, BroadPeak	Samtools, bamCoverage	Mapping results,peak calling results,motif finding, Go annotation,pathway analysis	findMotifsGenome.pl	annotatePeaks.pl	annotatePeaks.pl	iPAGE

The major contributions of CSA include 1) CSA integrates more comprehensive functions, from mapping to downstream analysis, and the tools used to convert formats are also integrated; 2) CSA provides a guideline for users to choose appropriate tools, and allows users to define their own workflows, which can help them complete their analysis through several easy steps; 3) CSA also provides the visualization of the entire process, including the results of mapping, peak calling, motif finding, and pathway analysis.

## Implementation

**Fig. 1:**
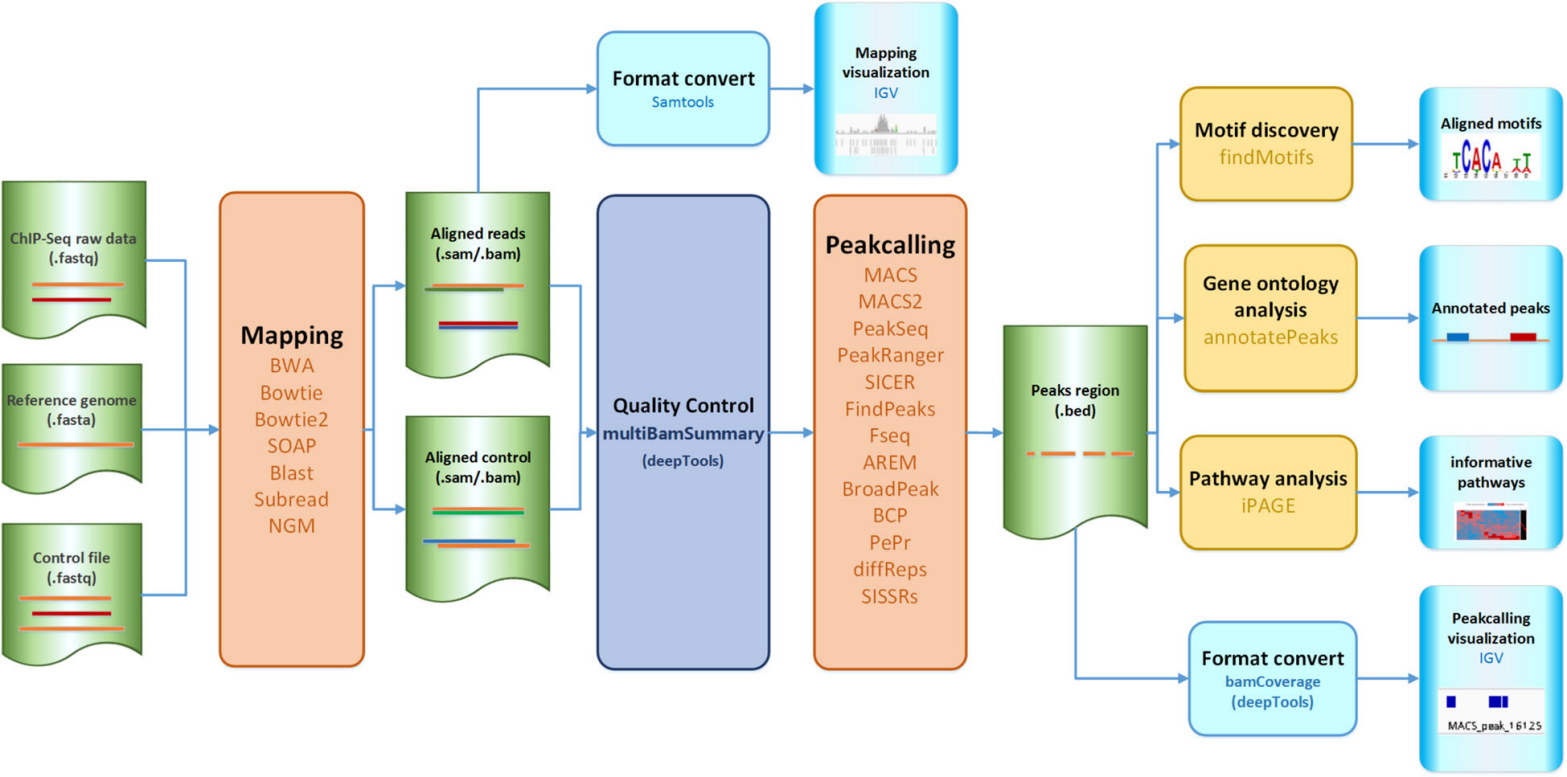
The pipeline of CSA, which includes four stages: mapping, quality control, peak calling and downstream analysis. Samtools and bamCoverage are used to convert the format. The visualization of mapping, peak calling, and downstream analysis are also provided

CSA provides the whole process of ChIP-seq analysis, and the pipeline of CSA for analyzing ChIP-seq data is shown in Fig. 1. In this pipeline, we take ChIP-seq raw data, a reference genome, and a control file as inputs. The step of mapping aligns short reads to reference sequences. Seven popular mapping tools: BWA [12], Bowtie [13], Bowtie2 [14], SOAP [15], BLAST [16], Subread [17], and NGM [18] are integrated in CSA. After mapping, CSA provides the step of quality control to check the correlation between replicates and published datasets by integrating multiBamSummary. Peak calling is the most important step which finds the enrichment of peak regions. Thirteen peak calling methods: MACS [19], MACS2, PeakSeq [20], PeakRanger [21], SICER [22], FindPeaks [15], Fseq [16], AREM [17], BroadPeak [18], BCP, PePr [19], diffReps [20], and SISSRs [29] are integrated in CSA. Moreover, three downstream analysis tools are integrated for motif analysis, GO analysis, and pathway analysis, to help users conduct further analysis and discover interesting results behind these data.

### Mapping and quality control

Mapping aligns short reads to long reference sequences, and is the most computationally intensive step in the overall data analysis process. Therefore, it is important to select the appropriate alignment strategy in this step. CSA integrates seven mapping tools, while each tool has its own advantages and disadvantages. To our best knowledge, no software systems can be applied to all cases. These tools are broadly based on two approaches: hash table and Burrows- Wheeler. Burrows-Wheeler is more common, and several tools, like BWA [12], Bowite [13], and SOAP [15], have been developed based on Burrows- Wheeler indexing. If the length of reads is greater than 100 bp, it’s better to use BWA. If the reads is short and single-end, Bowtie would get high accuracy. In addition, SOAP is suitable for both single-end and paired-end alignment, it reduces the usage of computer memory and improves the speed of processing reads.

Quality control is performed by the method of multiBamSummary, which is involved in the package of deepTools [38]. This tool is useful to find the correlation between published data sets and the files generated by the step of mapping. The result of this tool is an array of correlation coefficients which are displayed as a clustered heatmap. Users can judge how “strong” the relationship between the published data set and their own files.mapping and quality control.

### Peakcalling

Peakcalling detects the enrichment of peak regions in ChIP-seq analysis, and thirteen methods are integrated. SAM or BAM files generated by mapping along with the control file used as the input of Peakcalling. Peak signals are generally classified into three categories according to the shape of peaks and the type of raw data. These three types are: sharp, broad and mixed. The sharp peak signals usually presented at the protein-DNA binding sites or on the histone modification sites of the regulatory elements. The broad type of peak signals generally has relationship with transcription factors and the histone modification in the gene expression region. Most current tools are suitable for the analysis of sharp peaks, such as MACS [19]. In addition, SICER [22] is designed for broad peaks [39].

### Downstream analysis

We implemented three downstream analysis modules: motif analysis (findMotifs), GO analysis (annotatePeaks), and Pathway analysis (iPAGE [34]). Motif analysis module uses the BED file as input, and finds out whether the identified binding sites defined by the BED file contain the previously established consensus binding sequences for the respective proteins. Gene Ontology analysis module looks for the enrichment of various genomic annotations in peaks or regions described in the BED files. Pathway analysis module results in a *P*-value heatmap with significant categories.

### Visualization

Visualization provides users with display of sequence and peak distributions. CSA integrates IGV [40] to show the results of mapping and Peakcalling. After mapping, users can get SAM format files and the alignments of reads could be visualized with these files. In the figure of alignment, the gray arrows represent reads, while the arrow indicates the orientation of the mapping. The nucleotides marked in different colors indicate mismatches between the reads and the reference. Light gray areas and white blocks display the alignments. After Peakcalling, users can get the reports about the enrichment of peaks in which a BED file is involved. IGV [40] could display the regions of enrichment through the BED file. In the figure of Peakcalling, the blue lines represent the peaks, and the length of blue lines indicates the width of peaks.

## Results

### Case study 1: genome-wide co-localization of several transcription regulators on enhancers

This case study describes the approach reported in Nature Cell Biology [41]. We just perform the mapping and peak calling part of their ChIP-seq analysis. YAP and TAZ are potent inducers of cell proliferation, regulating organ growth and tumorigenesis. In their analysis, YAP and TAZ antibodies were used to perform the ChIP-seq experiment in MDA-MB-231 breast cancer cells. A list of tools were used for analysis, uniquely mapped reads were retained by using Bowtie [13] (version 0.12.7), and the reference genome was hg19. Samtools was used to remove the redundant reads. IDR (Irreproducible Discovery Rate) framework was used to evaluate the consistency of the replicate experiment. Peaks were detected by MACS2 version 2.0.10, and IgG ChIP-seq was used as the control sample. The IDR threshold of 0.01 was regarded as the standard to identify the best peaks number for all datasets. At last, the enrichment of each peak could be displayed by using IGV [40].

Preparing the input data file. Here we used the “WorkFlow” module to repeat this analysis process. Firstly, ChIP-seq dataset was downloaded from Gene Expression Omnibus (GEO) [42] with accession number of GSE66083. We can get the raw sequences of YAP/TAZ/TEAD/IgG in the format of SRA, and all these data files should be converted into FASTQ format by sratoolkit so that the files could fit the input format of “WorkFlow” module.

**Fig. 2:**
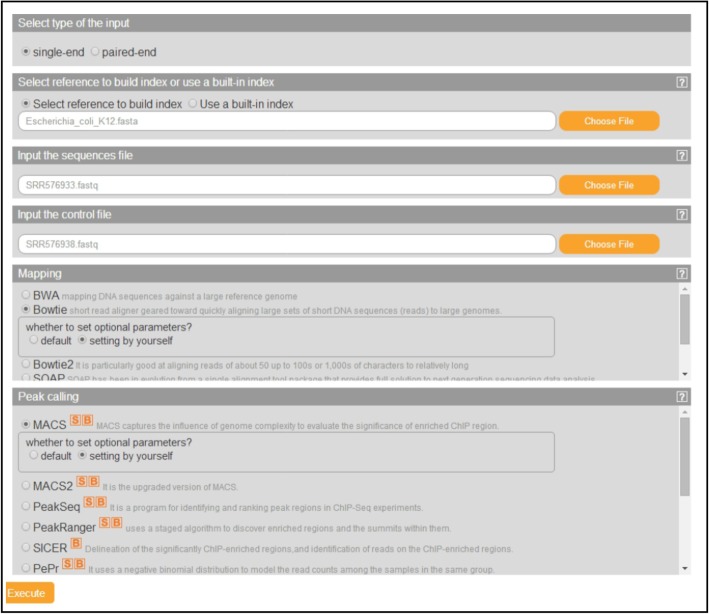
The definition of the workflow

Performing “WorkFlow”. On the page of “WorkFlow”, we selected “single-end” as the type of input, and then chose the sequences file of YAP in the format of FASTQ. CSA contained the references of genome hg19 and hg38, the reference was built in advance to save time, we clicked “Use a built-in index” to select the hg19 as the reference. In the field of control files, the FASTQ file of IgG should be input here. The mapping box contained 7 alignment tools integrated in CSA, here we chose Bowtie, and used the default parameters. The peak calling box contained 13 peak detection tools, we chose MACS2, and also used the default setting. The last step, after clicking the “Execute” button, the workflow started. We repeated the steps for the analysis of TAZ and TEAD. The definition of the workflow is shown in Fig. 2.

Viewing the output. When the operation was finished, the web jumped to the page of “Results visualization”. We learned from the analysis of Zanconato et al. that the region of promoters and enhancers here were defined by the genomic locations and overlaps of H3K4me1 and H3K4me3 peaks [43]. We selected one promoter region, and one enhancer region. Filling the file input field of scope with “chr4:41,518,010-41,541,509”, it took a while for the visualization tool to deal with the scope. After processing, the graph of peaks binding to promoters would display on the page, and users can also download the result files through the web page in one month. Additional file 1: Fig. S1. (A) in the supplementary material shows YAP/TAZ/TEAD binding to promoters with the scope of “chr4:41,518,010-41,541,509”. Then we input the scope of “chr4:41,118,180-41,141,679” to view the peaks binding to enhancers. Additional file 1: Fig. S1. (B) in the supplementary material shows YAP/TAZ/TEAD binding to enhancers. We recommend using “Mapping visualization” to view the enrichment. Because the visualization of peak calling is based on the bed format file, peaks are described with a lot of blue horizontal lines. Although we can get the number and the region of peaks from this graph, it is still not clearly to identify the correlation between transcriptomes.

We performed the analysis of YAP, TAZ, and TEAD separately, and input two scopes mentioned above for these three transcription factors independently. During these analysis, CSA generated 6 figures totally. For each transcription factors, two figures were created and represented binding to promoters and enhancers respectively. In order to compare these results more obviously, we intercepted the core region of these figures, and spliced them together, as shown in Additional file 1: Fig. S2.

From this case, we carried out genome-wide analyses of YAP/TAZ-binding sites through ChIP-seq, and found that most YAP/TAZ-bound cis-regulatory regions coincided with enhancer elements, located distant from TSSs. This finding can help researchers to capture new and essential aspects of YAP/TAZ mediated transcriptional regulation.

### Case study 2: motif discovery in ChIP-seq peaks

In this case, we used the dataset obtained from the study of Nature Cell Biology [41], which was described above. In their research, motif finding was crucial to find the correlation between variant transcription factors. The De novo motif finding and known motif finding were operated by the tool of findMotifs in HOMER [30]. In this study, 500 bp windows were used to search the motifs at the peak summits. The enrichment of known motifs was detected by screening the reliable motifs in HOMER motif database [44] and JASPAR database [45].

Data acquisition and processing. We reproduced the motif discovery following the method integrated in the CSA. The analysis processes were as follows. First, Supplementary Table 1 from Zanconato et al. was downloaded, the shared YAP/TAZ and TEAD4 binding sites. Second, the forth column (Chromosome), sixth column (start position), and seventh column (end position) were collected into a text file called “peak_mix.bed”. Then we used this file as the input of CSA, the appropriate genome should be hg19, and we used the default region size for motif finding: 200, and the optional parameters were chosen with the default setting.

Results visualization. Although several files were generated, here we concentrated on homerResults.html (showing the output of de novo motif finding in the form of web pages) and knownResults.html (showing the output of known motif finding in the form of web pages). From the page of homerResults.html, as shown in Additional file 1: Fig. S3, 18 de novo motifs were found, and there were two possible false positives, and motifs were ranked in accordance with the *p*-value in ascending order. The detail information of each motif was obtained by clicking the link “More Information”. On the detail information page, as shown in Additional file 1: Fig. S4, the logo of the motif and several numerical metrics were presented, and top ten known motifs that match best to this motif were listed, where the discovered de novo motif can be compared with the known motif database. Known motif databases here are the HOMER motif database and JASPAR database. From the page of knownResults.html, we can view the known motif discovery. Different from the known motifs found on the detail information page mentioned above, the known motifs here were found by comparing the regions that were contained in the bed format file to the known motif database. In addition, we also take GO enrichment analysis and KEGG pathway analysis, the results figures are shown in Additional file 1: Figs. S5 and S6.

## Conclusion

In this study, we have presented the CSA web server for the whole process of ChIP-seq analysis, including the step of mapping, quality control, peak calling, and downstream analysis. CSA also provides the function of workflow, which allows users to define their own procedure. In addition, CSA visualizes mapping, peak calling and motif finding results. For the common type of ChIP-seq datasets, including histone modifications and transcription factor, CSA can provide the corresponding tool for processing them. In addition, CSA can detect differences in ChIP signals between ChIP samples and controls to identify absolute binding sites. What’s more, for general ChIP-seq analysis, biologists need to perform multiple analysis steps, and each step needs different tools. Switches between different tools may take a lot of time for biologists to learn the usage of tools and convert the formats of data. Here we provide the modular design of workflows in CSA, through which users only need to provide raw data files, and select the appropriate tools and parameters, CSA can complete data analysis automatically.

## Supplementary information

**Additional file 1: Fig. S1.** YAP/TAZ/TEAD (A) binding to promoter; (B) binding to enhancers. **Fig. S2.** YAP/TAZ/TEAD binding comparison of promoters and enhancers. **Fig. S3.** CASE STUDY 2: Motif discovery in ChIP-Seq peaks. Finding de novo motifs. **Fig. S4.** CASE STUDY 2: Motif discovery in ChIP-Seq peaks. Detail information about the motif 1. **Fig. S5.** CASE STUDY 2: GO enrichment analysis for ChIP-Seq peaks. **Fig. S6.** CASE STUDY 2: KEGG pathway analysis for ChIP-Seq peaks.

## Data Availability

The supplementary materials are provided, and the website of CSA is available at http://CompuBio.csu.edu.cn. The datasets used in case study are available in accession GSE66083.

## Abbreviations

ChIP-seq: Chromatin immunoprecipitation sequencing
IDR: Irreproducible Discovery Rate
NGS: Next generation of sequencing technology
