# Supplementary material for: CSA: a web service for the complete process of ChIP-Seq analysis
Source: BMC Bioinformatics. 2019 Dec 24;20(Suppl 15):515. doi: 10.1186/s12859-019-3090-0 (PMC6929326; doi:10.1186/s12859-019-3090-0)
Supplement: Supplementary file 1 — Additional file 1: Fig. S1. YAP/TAZ/TEAD (A) binding to promoter; (B) binding to enhancers. Fig. S2. YAP/TAZ/TEAD binding comparison of promoters and enhancers. Fig. S3. CASE STUDY 2: Motif discovery in ChIP-Seq peaks. Finding de novo motifs. Fig. S4. CASE STUDY 2: Motif discovery in ChIP-Seq peaks. Detail information about the motif 1. Fig. S5. CASE STUDY 2: GO enrichment analysis for ChIP-Seq peaks. Fig. S6. CASE STUDY 2: KEGG pathway analysis for ChIP-Seq peaks. [file 12859_2019_3090_MOESM1_ESM.docx]

**Figure S1 - YAP/TAZ/TEAD (A) binding to promoter; (B) binding to enhancers.**

**A B**


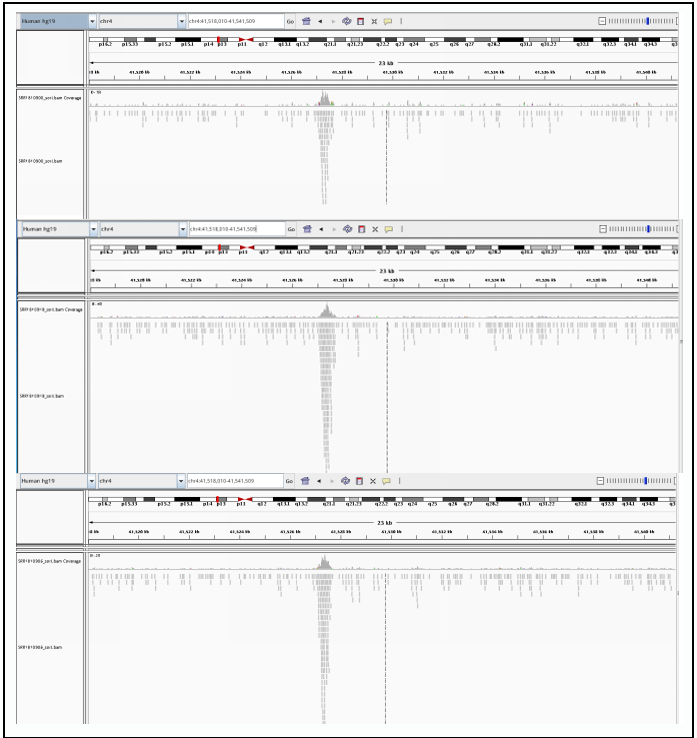

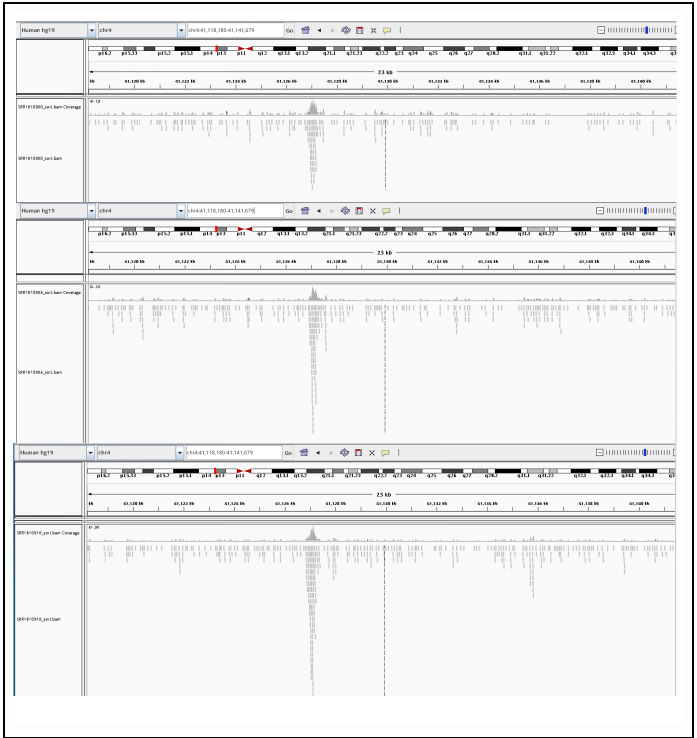


**Figure S2 - YAP/TAZ/TEAD binding comparison of promoters and enhancers.**


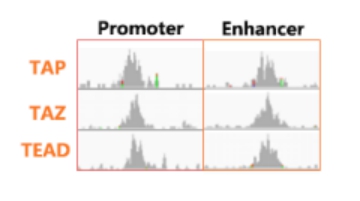


**Figure S3 - CASE STUDY 2: Motif discovery in ChIP-Seq peaks. Finding de novo motifs.**


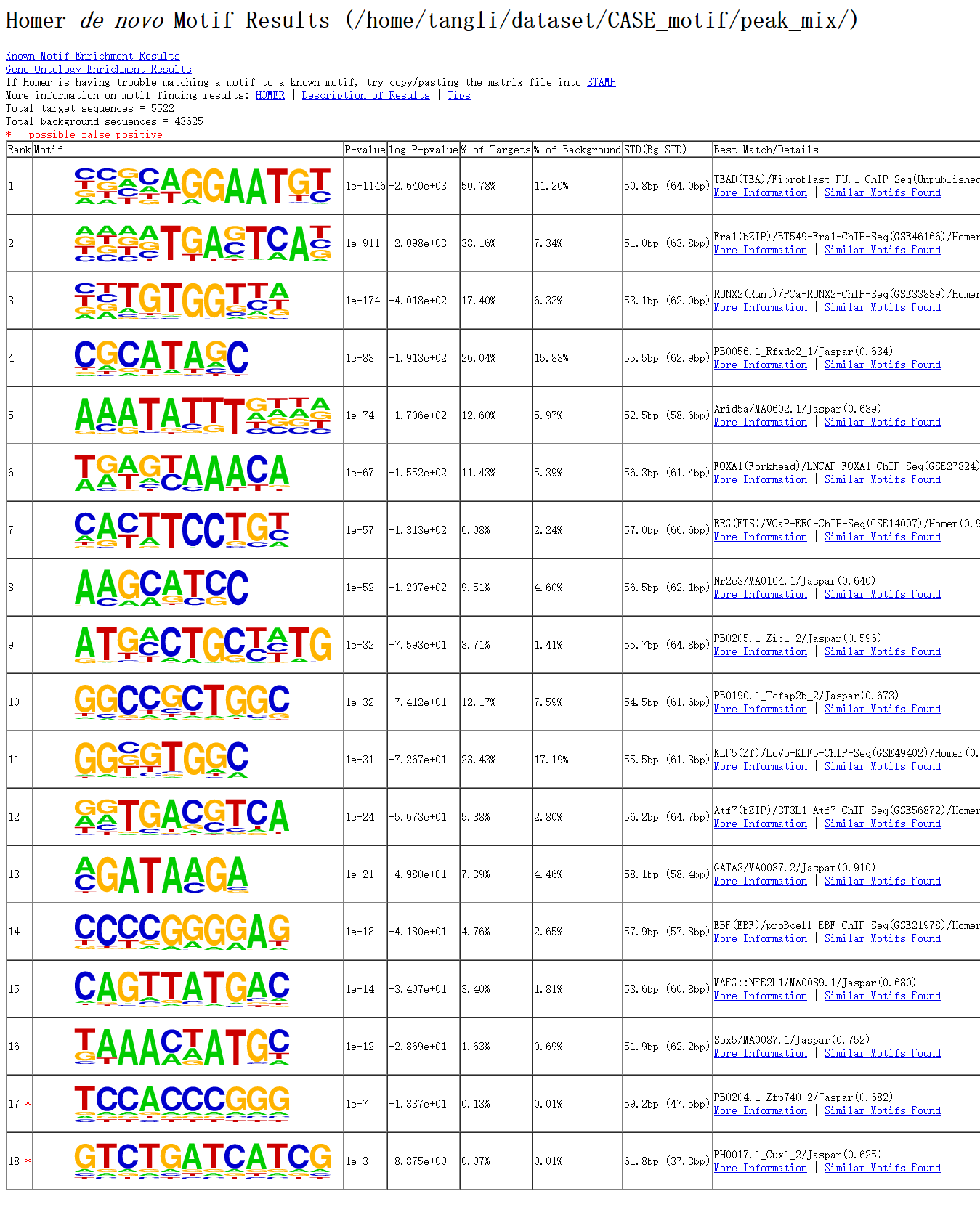


**Figure S4 - CASE STUDY 2: Motif discovery in ChIP-Seq peaks. Detail information about the motif 1.**


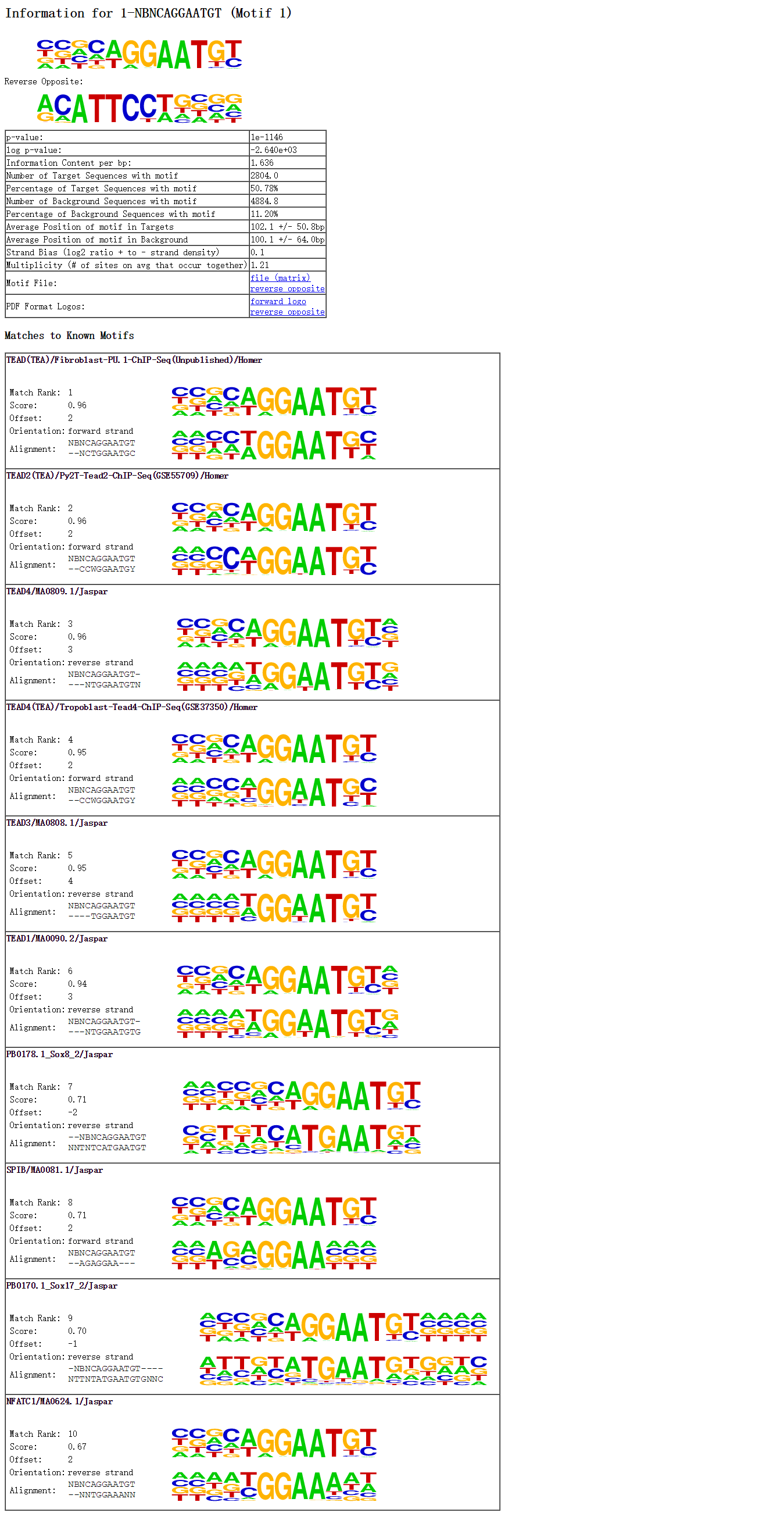


**Figure S5 - CASE STUDY 2: GO enrichment analysis for ChIP-Seq peaks.**

**Figure S6 - CASE STUDY 2: KEGG pathway analysis for ChIP-Seq peaks**
